# Transoral Biopsy of the Clivus in a Pediatric Patient for Suspected Osteomyelitis

**DOI:** 10.7759/cureus.29548

**Published:** 2022-09-25

**Authors:** Ethan Davoudzadeh, Kan Chen, Faizullah Mashriqi, Drew M Caplin

**Affiliations:** 1 Interventional Radiology, Donald and Barbara Zucker School of Medicine at Hofstra/Northwell, New Hyde Park, USA

**Keywords:** grisel syndrome, interventional radiology, clivus, osteomyelitis, skull base, transoral biopsy

## Abstract

Pediatric skull base osteomyelitis is an uncommon and difficult infection to characterize and treat, and it can result in devastating neurologic sequela. While transoral biopsy of the clivus in the adult population has been demonstrated, no such case is reported in the literature for the pediatric population for the purposes of elucidating an infectious source. Here we describe transoral biopsy of the clivus utilizing computed tomography (CT) guidance in a pediatric patient with suspected skull base osteomyelitis.

## Introduction

Osteomyelitis involving the clivus and basion-dens interval is a rare phenomenon in the pediatric population, accounting for 1-2% of all cases of osteomyelitis [1]. Several different organisms, including those of bacterial, viral, fungal, and parasitic origins, may cause such an infection, making the treatment algorithm difficult. Oftentimes, diagnosis is delayed due to the nonspecific presentation and laboratory/imaging results, leading to severe consequences such as the spread of infection and devastating neurological sequelae [2]. Thus, safe techniques to obtain tissue for organism identification to allow for culture-guided antibiotic management are imperative for proper patient care [3].

## Case presentation

**Figure 1:**
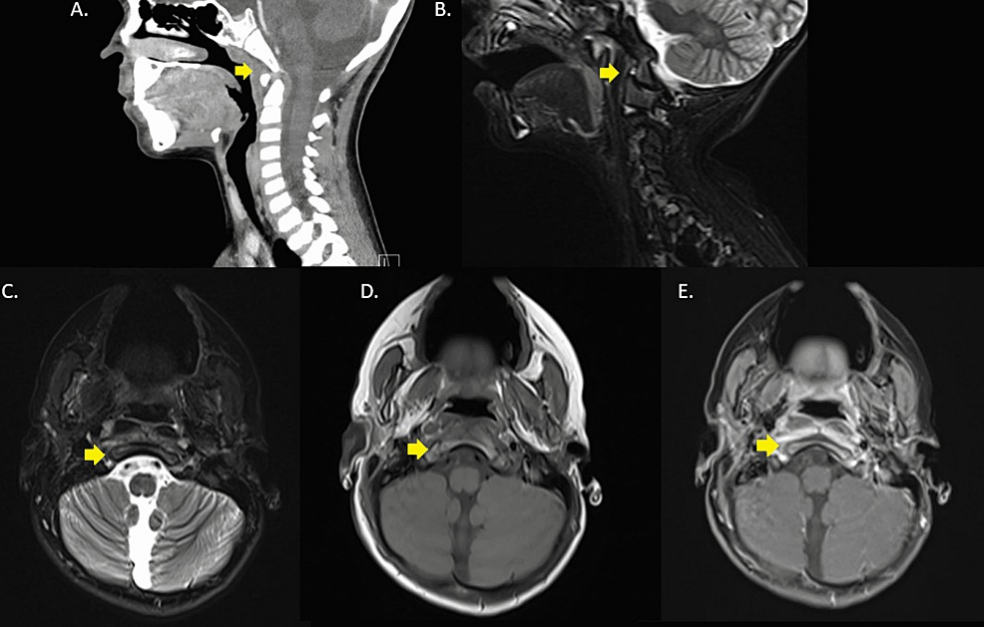
Grisel syndrome (A) Sagittal section contrast-enhanced CT of the neck; (B) Sagittal section T2-weighted image with fat suppression of the cervical spine; (C) Axial section T2-weighted image with fat suppression of the skull base; (D) Axial section T1-weighted image pre-contrast of the skull base; (D) Axial section T1-weighted image post-contrast of the skull base.

A five-year-old male with a past medical history of global developmental delay presented to his pediatrician with one month of neck pain, nausea, and vomiting. The neck pain was intermittent, associated with torticollis, and resulted in decreased oral intake. The patient's parents endorsed a fever of 102.7F prior to the presentation. The pain and fever were managed with over-the-counter analgesics at home. There was no reported trauma or neurological deficits. The patient did not recently travel and had no known sick or animal contacts. His initial vital signs were within normal limits. On physical exam, the only pertinent positive involved difficulty opening the mouth. There was no lymphadenopathy. Lab work on presentation showed normal leukocyte count and an elevated erythrocyte sedimentation rate (ESR) of 21. Initial outpatient cervical spine radiographs were unremarkable. CT of the neck soft tissues revealed left maxillary sinus opacification (not shown). Lastly, an outpatient MRI of the cervical spine and neck demonstrated thickening and enhancement involving the prevertebral soft tissues of the upper cervical spine, associated bone marrow edema, and atlantoaxial malalignment. This constellation of findings was suspicious for Grisel syndrome (Figure 1). As a result, the patient was started on a 14-day course of amoxicillin-clavulanic acid and was referred for a neurosurgical evaluation. 

**Figure 2:**
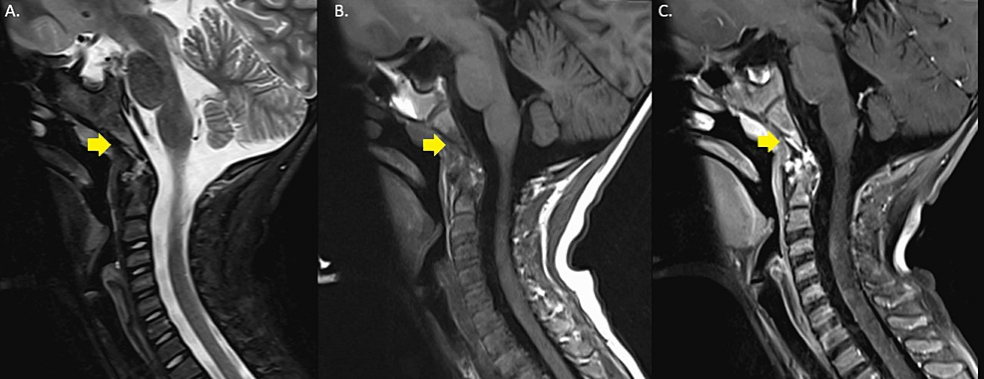
Skull base osteomyelitis (A) Sagittal section T2-weighted image with fat supression of the cervical spine; (B) Sagittal section T1-weighted image pre-contrast of the cervical spine; (C) Sagittal section T1-weighted image post-contrast of the cervical spine.

Despite the antibiotic regimen, the patient's symptoms persisted and worsened. Five weeks from the initial presentation, the neurosurgeon ordered repeat bloodwork and cervical spine MRI. Bloodwork demonstrated an increasing ESR from 21 to 44. The MRI showed bone marrow edema and enhancement involving the occipital condyles, lateral masses of C1 and C2, clivus, and ligaments between C1/C2, concerning for osteomyelitis (Figure 2). At this time, the patient was instructed to go to the emergency department for further workup and treatment. In the emergency department, the patient's vitals were within normal limits. The physical exam demonstrated cervical spine tenderness. Repeat labs were without significant interval change. Blood cultures were negative after five days, and all viral serologies were also negative. 

Interventional radiology was consulted to perform a biopsy for culture sensitivities to help guide antibiotic management. After a multidisciplinary discussion with pediatrics, infectious disease, otolaryngology, and neurosurgery, a decision was made to perform a transoral biopsy of the clivus as an open biopsy would be associated with increased morbidity. Given the clinical history, MRI findings, and physical exam findings of cervical spine tenderness, the skull base biopsy was thought to be more appropriate than a lumbar puncture to assess for an infectious etiology.

After a thorough pre-procedure evaluation of the patient and obtaining informed consent from the parents, the patient was placed supine on the CT table, and endotracheal intubation was performed by the anesthesiologist. A tongue depressor and dental guard were used as a bite block and tongue retractor to facilitate the transoral approach. The mouth and oropharynx were disinfected with Betadine.

**Figure 3:**
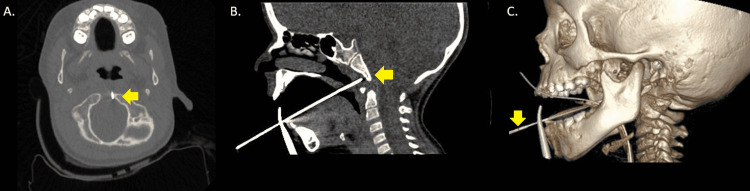
Transoral biopsy of the clivus (A) Axial intraoperative CT at the level of the foramen magnum; (B) Sagittal intraoperative CT at the midline; (C) Intraoperative 3D reconstruction.

Once the oral aperture was optimized, a 19-gauge introducer needle with the blunt stylette was positioned against the posterior pharyngeal wall and secured with a clamp, after which a CT of the upper cervical spine was performed. Once the angle and location of the needle were confirmed, the sharp stylette was inserted, and the introducer needle was advanced through the posterior pharyngeal soft tissue. Multiple fine needle aspiration (FNA) samples were obtained from the clivus as well as the basion-dens interval using a coaxially inserted 22-gauge Chiba needle (Figure 3). The needles were removed, and hemostasis was achieved with direct pressure. Post-procedure imaging was performed.

The patient tolerated the procedure well, and there were no procedure-related complications. The patient was then transferred to the pediatric intensive care unit for observation and monitoring. The aspirated sample yielded methicillin-susceptible *Staphylococcus aureus* and alpha-hemolytic *Streptococcus*. Cytology demonstrated benign squamous cells with scattered benign glandular epithelium. At this time, the patient was started on ceftriaxone and nafcillin for a four to six-week time course via a peripherally inserted central catheter (PICC). On a one-month follow-up visit to the infectious disease clinic, the patient and the patient's parents report complete resolution of pain and a return to a normal range of motion. 

## Discussion

Osteomyelitis of the skull base is an uncommon occurrence in the pediatric population. In developed nations, skull base osteomyelitis is seen in the postoperative setting. In underdeveloped nations, paranasal sinus disease, trauma, and scalp infections are more common causes [3]. *Pseudomonas aeruginosa* is the causative organism of skull base osteomyelitis in 90-98% of cases [3]. Additional causes included S*taphylococcus* and *Salmonella *species and fungi, including *Candida *species, *Aspergillus*, and *Cryptococcus *[3].

The nonspecific presentation of skull base osteomyelitis makes its diagnosis difficult. Patients may complain of ear pain and loss of hearing if the cause of the skull base osteomyelitis is secondary to malignant otitis. Facial nerve involvement is also common in lateral cases of skull base osteomyelitis [3]. More medially, abducens nerve palsy may result from the spread of infection toward the brainstem [3]. Headaches and fevers are additional nonspecific presenting signs [3]. Delayed diagnosis and treatment can result in devastating consequences, including the spread of infection to the intracranial compartments. Therefore, identifying the underlying organism to guide antibiotic management is key to successful treatment. Skull base osteomyelitis poses a unique challenge in identifying this causative organism due to the difficulty in obtaining a biopsy.

We have discussed a transoral biopsy technique for osteomyelitis involving the clivus in which a culture identifying the culprit organisms was obtained, and proper antibiotics were administered, with a resolution of symptoms. The transoral technique has been reported as a safe technique in the past, specifically in the setting of vertebroplasty or biopsies of soft tissue masses in the upper cervical region [4,5]. One of these cases was in the pediatric population and involved a five-year-old female presenting with neck stiffness and a mass extending from the clivus to C2 on CT and MRI. The authors here used a similar transoral technique to biopsy the mass which was diagnosed as a chordoma [5]. To our knowledge, there is no literature on the use of this transoral technique in the pediatric population to elucidate the cause of skull base osteomyelitis. Our case shows that the transoral technique is a direct method for evaluating infections of the clivus and basion-dens interval that requires extensive multidisciplinary discussion, careful patient evaluation, and procedure setup for optimal results.

## Conclusions

Pediatric skull base osteomyelitis is a difficult infection to characterize and treat, often requiring a multidisciplinary approach involving pediatrics, infectious disease, neurosurgery, anesthesiology, and interventional radiologists. While transoral biopsy of the clivus, a safe procedure, has been demonstrated in the adult population, its use in the pediatric population has not yet been reported. This case report shows a successful transoral approach in a pediatric patient.
